# Interferon-α enhances the susceptibility of renal cell carcinoma to rapamycin by suppressing mTOR activity

**DOI:** 10.3892/etm.2014.1691

**Published:** 2014-04-25

**Authors:** XIAO HAN, DONGHAO SHANG, TIANDONG HAN, XIUHONG XU, YE TIAN

**Affiliations:** Department of Urology, Beijing Friendship Hospital, Capital Medical University, Beijing 100050, P.R. China

**Keywords:** renal cell carcinoma, interferon-α, rapamycin, mammalian target of rapamycin

## Abstract

The aim of the present study was to investigate the antiproliferative effects of interferon (IFN)-α and rapamycin (RPM) on renal cell carcinoma (RCC) cells and examine the synergistic growth suppression conferred by IFN-α and RPM. The effects of IFN-α and/or RPM on RCC cells were determined using a WST-1 assay and the synergy of IFN-α and RPM against three RCC cell lines was analyzed with isobolographic analysis. The expression of mammalian target of rapamycin (mTOR) was downregulated by RNAi, and the expression and phosphorylation of proteins in the mTOR pathway following treatment with IFN-α and/or RPM was examined by western blot analysis. The observations indicated that IFN-α significantly increased the susceptibility of RCC cells to RPM and the synergistic effect of IFN-α and RPM against RCC cells was confirmed in all three RCC cell lines. The mTOR pathway was shown to be associated with the synergistic effect of IFN-α and RPM against RCC. IFN-α and RPM alone decreased the phosphorylation of mTOR, p70 S6 kinase, S6 and 4E binding protein 1, and IFN-α significantly enhanced the RPM-induced suppression of the mTOR pathway. However, in RCC cells with low mTOR activity, the synergy of IFN-α and RPM was eliminated. Therefore, the results of the present study indicate that the mTOR pathway plays an important role in the synergistic effect of IFN-α and RPM against RCC cells. Thus, mTOR may serve as an effective therapeutic target in the treatment of advanced RCC.

## Introduction

Renal cell carcinoma (RCC) is the most common type of kidney cancer in adults. Following the occurrence of metastasis, survival rates are very poor and the 5-year survival rate is ~20% (1). RCC is resistant to chemotherapy (2). At present, treatment regimens using interferon (IFN)-α have been applied in clinical practice to treat RCC, achieving therapeutic response rates between 4 and 33% (3). A previous study revealed that IFN-α mediates anticancer effects indirectly by modulating immunomodulatory mechanisms or directly through antiproliferative effects and inducing the differentiation of cancer cells (4).

IFN-α exerts these effects by binding to cell surface receptors and activating the Janus kinase (Jak) protein family. Activated Jak1 and tyrosine kinase 2 phosphorylate signal transducers and activators of transcription (STATs). Subsequently, phospho-STATs translocate to the nucleus and interact with specific regulatory elements to induce target gene transcription (5). RCC treatment has developed significantly, as vascular endothelial growth factor (VEGF) receptor tyrosine kinase inhibitors and drugs that inhibit mammalian target of rapamycin (mTOR) signaling have become the mainstay for the management of advanced RCC. These treatments have improved progression-free survival and/or overall survival outcomes (6). The mTOR pathway has been reported to be central to cancer progression and rapamycin (RPM) has been shown to suppress carcinogenesis by decreasing mTOR activity (7). RPM may function by stimulating the degradation of cyclin D1, which inhibits the G1 to S-phase transition in the cell cycle (8). RPM also downregulates phospho-p70 S6 kinase (K), which is considered to be an indicator of the activated mTOR pathway (9). The primary substrate of p70 S6K, S6 ribosomal protein, has also been shown to have an important role in determining cell size. Phosphorylation of the eukaryotic translation initiation factor, 4E binding protein 1 (4E-BP1), by mTOR results in the activation of cap-dependent translation of nuclear mRNAs by releasing the inhibition of the eukaryotic translation initiation factor 4E (10). RPM has been shown to suppress the growth of small cell lung cancer and pancreatic cancer cells (11,12). In addition, mTOR inhibitors have shown promising efficacy in early-stage trials in patients with advanced RCC (13). A previous study indicated that RPM may be of value to patients with RCC and that the antitumor efficacy of RPM is achieved by cell-cycle arrest and targeted reduction of VEGF-A and transforming growth factor-β1 (14). An additional study revealed the synergistic effects of RPM and chemotherapeutic agents against tumor cells; RPM was reported to increase the cytotoxicity of cisplatin by sensitizing human promyelocytic leukemia and ovarian cancer cells to the drug, thereby inducing apoptosis (15). However, receptor tyrosine kinase inhibitors only demonstrate additive effects in combination with RPM in the treatment of prostate cancer (16). A previous study indicated that IFN-α suppresses the phosphoinositide 3 kinase and mTOR signaling pathways (17). Furthermore, combining RPM with other upstream mTOR inhibitors has been shown to induce greater growth suppression in RCC compared with that achieved by administering the drugs alone (18). However, whether IFN-α and RPM have a synergistic effect against RCC remains unknown.

High frequency mutations or the loss of the two copies of the Von Hippel-Lindau (VHL) tumor suppressor gene have been observed in RCC (19). VHL protein is the substrate recognition component of the E3 ligase that ubiquitinates hypoxia-inducible transcription factors (HIFs), including HIF-1α and -2α. VHL plays a pivotal role in the downregulation of VEGF expression (20). Previous studies have indicated that mTOR stimulates HIF expression and RPM exhibits antiangiogenic activity that is associated with a reduction in the production of HIF/VEGF (21,22). However, the effect of VHL activity on the antiproliferative ability of IFN-α and RPM in RCC remains unknown.

## Materials and methods

### Cell lines and agents

Three RCC cell lines, ACHN, NC65 and A498 (ATCC, Rockefeller, MD, USA), were cultured in complete medium consisting of RPMI-1640 (Gibco, Gaithersburg, MD, USA) supplemented with 25 mM hydroxyethyl piperazineethanesulfonic acid, 2 mM L-glutamine, 1% nonessential amino acids, 100 U/ml penicillin, 100 μg/ml streptomycin and 10% heat-inactivated fetal bovine serum. Cell lines were maintained as monolayers on 10-cm plastic dishes and incubated in a humidified atmosphere containing 5% CO_2_ at 37°C. Intron A (recombinant IFN-α2b) was purchased from Merck & Co, Inc. (Whitehouse Station, NJ, USA) and RPM was purchased from Sigma-Aldrich (St. Louis, MO, USA).

### WST-1 assays

Effects of IFN-α and/or RPM on the RCC cells were determined using a WST-1 assay. Exponentially growing cells were harvested and seeded at 2,000 cells/well in a 96-well microtiter plate. After 4 h of incubation, Intron A (10, 50, 100, 200, 400 or 800 IU/ml), RPM (1, 5, 10, 15 or 20 μM), a combination of Intron A (50 or 100 IU/ml) and RPM (1, 5, 10, 15 or 20 μM), or penicillin/streptomycin medium (untreated control) were added. The cells were then continuously incubated for 72 h. WST-1 (Roche Diagnostics, Penzberg, Germany) at a volume of 10 μl was added to each well and the cells were incubated for an additional 2 h. Absorbance was measured with a microculture plate reader (Immunoreader; Japan Intermed Co., Ltd., Tokyo, Japan) at 450 nm. The percentage of cell cytotoxicity was calculated using the following formula: % Cytotoxicity = [1 − (absorbance of experimental − absorbance of blank)/(absorbance of untreated control − absorbance of blank)] × 100.

### siRNA transfection

A498 cells, which lack the wild-type VHL gene, were stably transfected using Lipofectamine 2000 (Invitrogen Life Technologies, Carlsbad, CA, USA) with an expression vector containing the full-length cDNA for VHL or with a blank vector without the VHL insert. Single colonies were selected with G418 and confirmed by cell staining, western blot analysis and cDNA sequencing. ACHN and A498 cells were seeded in complete medium without antibiotics and were allowed to grow until 30–50% confluence was reached. The cells were then transfected with siRNA oligonucleotides or scrambled siRNA control using Lipofectamine 2000. Following incubation for 72 h, gene expression was confirmed by western blot analysis. SignalSilence mTOR siRNA I was purchased from Cell Signaling Technology, Inc. (Beverly, MA, USA). All RNAi target sequences and oligonucleotide sets used in the study are shown in Table I.

### Reverse transcription polymerase chain reaction (RT-PCR)

Total RNA was isolated using an RNeasy mini kit (Qiagen, Frankfurt, Germany). A first-strand cDNA synthesis kit (GE Healthcare, Little Chalfont, UK) was used for reverse transcription. The PCR conditions were selected according to the manufacturer’s instructions and the expected sizes of the PCR products were confirmed by agarose gel electrophoresis. The PCR products were quantified with a GeneAmp 5700 Sequence Detection system (Applied Biosystems, Inc., Foster City, CA, USA). All primer sets used in this study are shown in Table I.

### Western blot analysis

The procedures were performed as previously described (23). Protein was extracted and the concentration was measured using a Bradford dye-binding protein assay (Bio-Rad Laboratories, Inc., Richmond, CA, USA). Subsequently, SDS polyacrylamide gel electrophoresis was performed. Anti-β-actin monoclonal antibodies (Abcam, Cambridge, UK) were used as an internal control. Other antibodies used in the study were all purchased from Cell Signaling Technology, Inc.. These included mTOR (7C10)/phospho-mTOR (Ser2481), p70 S6K (49D7)/phospho-p70 S6K (Thr421/Ser424), S6 ribosomal protein (5G10)/phospho-S6 ribosomal protein (Ser240/244) (D68F8) XP and 4E-BP1 (53H11)/phospho-4E-BP1 (Thr70) rabbit monoclonal antibodies. Immune complexes were detected using an enhanced chemiluminescence system (GE Healthcare) combined with image analysis. The image analysis software used was ImageJ (NIH, Bethesda, MD, USA).

### Statistical analysis

All determinations were performed in triplicate and the results are expressed as the mean ± SD. Statistical significance was determined using the Student’s t-test and P<0.05 was considered to indicate a statistically significant difference. Synergy was evaluated by isobolographic analysis, as described by Berenbaum (24). The fractional inhibitory concentration of each agent was equal to the IC_50_ dosage of the agent in combination divided by the IC_50_ dosage of the agent when used alone. An additive, synergistic or antagonistic combination was indicated by whether the point lies on, below or above, respectively, the straight line joining the dosages of the two drugs that when administered alone produce the same effect as that of the combination, as based on the isobolographic analysis.

## Results

### Synergistic growth suppression by IFN-α and RPM

**Figure 1:**
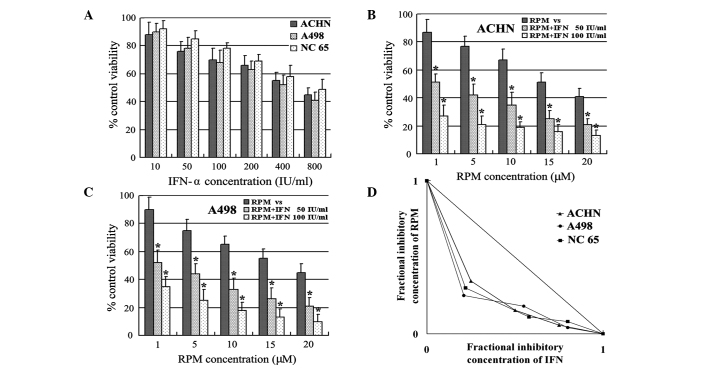
Synergy of IFN-α and RPM in RCC cell lines. Bar graphs showing the effect of (A) IFN-α on RCC cells, and the combination effect of IFN-α and RPM on (B) ACHN and (C) A498 cell proliferation. (D) Synergy of IFN-α and RPM in RCC cell lines was assessed by isobolographic analysis. All determinations were performed in triplicate and error bars represent SD. ^*^P<0.01 vs. RPM alone. IFN, interferon; RPM, rapamycin; RCC, renal cell carcinoma.

IFN-α administration caused dose-dependent cell growth inhibition in the ACHN, A498 and NC65 RCC cell lines (Fig. 1A). In addition, a combination of IFN-α and RPM caused dose-dependent cell growth inhibition in the RCC cell lines (Figs. 1B and C). IFN-α, at low concentrations of 50 and 100 IU/ml, significantly increased the susceptibility of the ACHN and A498 RCC cell lines to RPM (Fig. 1B and C). Combined treatment with IFN-α and RPM resulted in synergistic growth suppression in all the RCC cell lines examined in this study, as shown by isobolographic analysis (Fig. 1D).

### Suppression of mTOR pathway components by IFN-α and/or RPM

**Figure 2:**
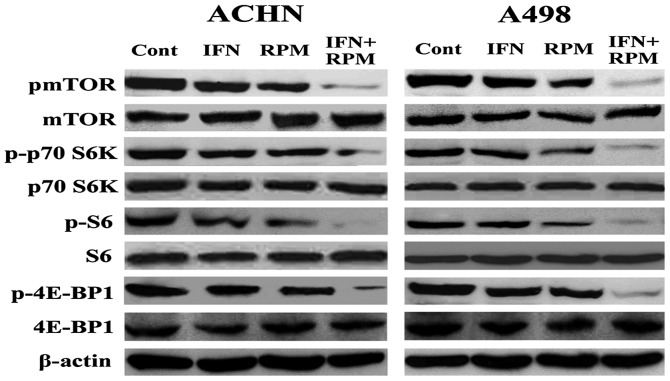
Suppression of the mTOR pathway by IFN-α and/or RPM. Western blot analysis demonstrates the effects of IFN-α and RPM, alone and in combination, on the ACHN and A498 RCC cell lines. mTOR, mammalian target of rapamycin; IFN, interferon; RPM, rapamycin; RCC, renal cell carcinoma.

To determine if the mTOR pathway is involved in the synergistic effect of IFN-α and RPM against RCC cells, phosphorylation of the mTOR pathway was evaluated following stimulation with IFN-α and/or RPM. In the ACHN and A498 cell lines, although 100 IU/ml IFN-α and/or 5 μM RPM did not affect the total protein expression of mTOR, p70 S6K, S6 or 4E-BP1, it was observed that IFN-α and RPM, alone or in combination, decreased the phosphorylation of mTOR, p70 S6K, S6 and 4E-BP1, as determined by western blot analysis (Fig. 2). In addition, IFN-α significantly enhanced the RPM-induced suppression of the mTOR pathway in these two cell lines. These results indicate that the mTOR pathway plays a key role in the synergistic effect of IFN-α and RPM against RCC cells.

### Effect of mTOR activity on the synergy of IFN-α and RPM

**Figure 3:**
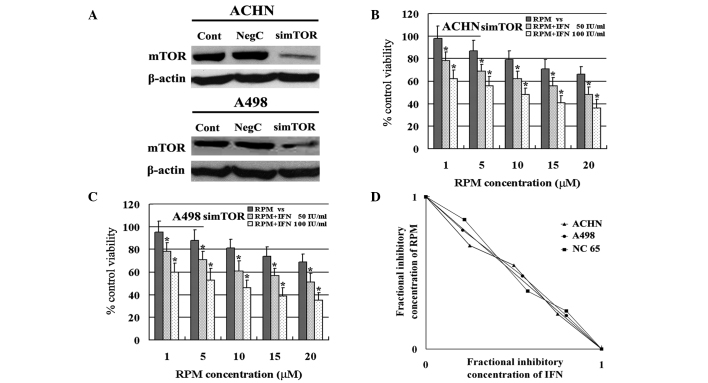
Effect of mTOR activity on the synergy of IFN-α and RPM. (A) Western blot analysis showing the expression of mTOR in two RCC cell lines transfected with RNAi. Bar graphs demonstrating the combination effect of IFN-α and RPM on m-TOR silenced (B) ACHN and (C) A498 RCC cells. (D) Effect of IFN-α and RPM on two m-TOR silenced RCC cell lines based on isobolographic analysis. All determinations were performed in triplicate and error bars represent SD. ^*^P<0.01 vs. RPM alone. mTOR; mammalian target of rapamycin; IFN, interferon; RPM, rapamycin; RCC, renal cell carcinoma.

The effect of mTOR activity on the synergy of IFN-α and RPM against RCC was investigated. The expression of mTOR was downregulated by RNAi and the results indicated that mTOR expression was suppressed effectively in ACHN and A498 cells (Fig. 3A). Regardless of mTOR expression, IFN-α enhanced the susceptibility of RCC to RPM in ACHN and A498 cells (Fig. 3B and C). However, the synergy of the two agents was eliminated in these cell lines, as an additive effect was indicated by isobolographic analysis (Fig. 3D). These results indicate that mTOR activity is necessary for the synergistic effect of IFN-α and RPM against RCC cells.

### Effect of VHL activity on the synergy of IFN-α and RPM

**Figure 4:**
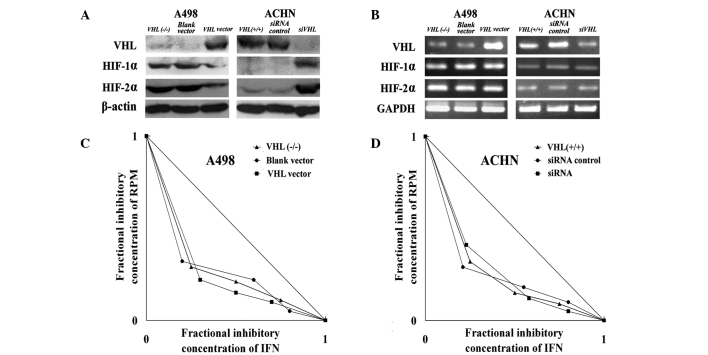
Effect of VHL activity on the synergy of IFN-α and RPM. VHL expression of VHL was downregulated in ACHN cells by RNAi and upregulated in A498 cells by transfection with a VHL vector, as confirmed by (A) western blot analysis and (B) RT-PCR. Isobolographic analysis demonstrated the effect of combination therapy with IFN-α and RPM on (C) A498 and (D) ACHN RCC cell lines. VHL, Von Hippel-Lindau; IFN, interferon; RPM, rapamycin; HIF, hypoxia-inducible transcription factor; RT-PCR, reverse transcription polymerase chain reaction; RCC, renal cell carcinoma.

The effect of VHL activity on the synergy of IFN-α and RPM against RCC was also investigated. VHL expression was downregulated in ACHN cells via RNAi and upregulated in A498 cells by transfection with a VHL vector. VHL/HIF expression was confirmed by western blot analysis and RT-PCR (Fig. 4A and B). Since VHL mediates HIF levels via a post-translational mechanism, VHL did not alter the mRNA expression levels of HIF-1α or -2α. Therefore, the results indicate that regardless of VHL activity, IFN-α enhances the susceptibility of RCC to RPM in all RCC cells tested in the study (Fig. 4C and D). Thus, the synergy of IFN-α and RPM does not depend on VHL activity in RCC cells.

## Discussion

Although a number of clinical trials with various combination chemotherapies have been performed in an attempt to overcome the current limitations of advanced RCC treatment, few have achieved favorable results or prognosis for patients with the disease (25). Therefore, the development of more effective combination chemotherapies for advanced RCC is required.

Promising new combination chemotherapies are usually identified simultaneously with advances in the understanding of oncogenesis. RPM has previously been reported to have immunosuppressant and anticancer effects on a large variety of malignancies, including hepatocellular carcinoma and RCC (26,27). In addition, RPM is well tolerated with minimal side-effects and has shown anticancer activity in patients with androgen-independent prostate cancer (28). Previous studies concerning combination chemotherapy of RPM with chemotherapeutic agents have been performed and combinations of RPM with bevacizumab, sorafenib or 5-fluorouracil have been reported to be promising therapeutic approaches for the treatment of hepatocellular carcinoma (29–33). IFN-α therapy is the most common approach for advanced RCC. However, the synergistic effects of RPM and IFN-α against RCC remain unclear. In the present study, the effect of a combination of IFN-α and RPM on the inhibition of RCC cell growth was analyzed. The results demonstrated that IFN-α and RPM caused dose-dependent inhibition of proliferation and combined treatment with the two agents resulted in synergistic growth suppression in all three RCC cell lines examined. At present, IFN-α is widely administered for the treatment of RCC. The observations of the present study indicate that RPM may be an optimal agent to combine with IFN-α for clinical application against RCC. Since chemotherapy is associated with severe side-effects that usually limit the clinical application, reducing the dosage of IFN-α or RPM is expected to alleviate the associated side-effects but not decrease the synergistic effects of these agents. Therefore, further clinical trials are required to analyze the tolerance towards IFN-α and RPM and to reveal the possible synergy of the two agents in patients with RCC.

The underlying mechanism behind the synergy between IFN-α and RPM in RCC cell lines was further investigated. The molecular mechanism promoting the anticancer effects of RPM is complex, as RPM suppresses the activity of mTOR and the phosphorylation of its downstream effectors, p70S6K and 4E-BP1 (34). The mTOR pathway is considered to be a central regulator in various malignant tumors. There are two distinct functional mTOR complexes. Firstly, mTORC1 consists of mTOR and regulatory-associated protein of mTOR (Raptor) and increases the phosphorylation of p70 S6K/4E-BP1. Secondly, there is mTORC2, which consists of mTOR and rapamycin-insensitive companion of mTOR and increases Akt (also known as protein kinase B) phosphorylation (35). Akt enhances cell growth by alleviating the tuberous sclerosis complex 1/2 suppression of mTOR, allowing the latter to function as part of the mTOR/Raptor complex on p70 S6K and 4E-BP1 (36,37). p70 S6K phosphorylates the S6 protein of the 40 S ribosomal subunit (38), while translation repressor protein 4E-BP1 inhibits translation by binding to the translation initiation factor eIF4E (39,40). Hyperphosphorylation of 4E-BP1 disrupts this interaction and results in the activation of translation (41).

In the present study, the role of the mTOR pathway in the synergistic effect of IFN-α and RPM against RCC was investigated. The results indicated that IFN-α and RPM did not affect protein expression in the mTOR pathway. However, each agent individually decreased the phosphorylation of mTOR, p70 S6K, S6 and 4E-BP1 in RCC cells. In addition, IFN-α significantly enhanced the RPM-induced suppression of the mTOR pathway, indicating that the synergy between IFN-α and RPM against RCC depends on the suppression of the mTOR pathway. The effect of mTOR activity on the synergy of IFN-α and RPM was also analyzed. In RCC cells expressing low levels of mTOR, the synergistic growth suppression of the two agents was eliminated and an additive effect was observed. These observations indicate that mTOR activity is important for the synergy of IFN-α and RPM against RCC cells.

Inactivation of the VHL tumor suppressor protein is a common event in clear cell RCC, which is the most common form of kidney cancer. A previous study reported that, in response to IFN-α, the exponential growth of wild-type VHL RCC cells was inhibited more than that of VHL-null RCC cells. This observation indicated that VHL inactivation may be involved in IFN-α resistance and that combined immunotherapy with antiangiogenic drugs may be beneficial for patients with a mutated VHL gene (42,43). However, the effect of VHL activity on the synergy of IFN-α and RPM against RCC is unknown. In the present study, A498 was used as the VHL-null RCC cell line, while the other cell lines were wild type for VHL. The results indicated that regardless of VHL activity, synergy of IFN-α and RPM was observed in all RCC cells and, thus, may be independent of VHL activity.

In conclusion, the present study demonstrated that the mTOR pathway plays an important role in the synergistic effect of IFN-α and RPM against RCC cells. The results indicate that blocking the activity of mTOR may provide a novel treatment strategy for patients with RCC. In addition, the suppression of RCC cell growth by IFN-α and RPM may be more effective in RCC cells with high mTOR activity.

## Figures and Tables

**Table I:** Primer and RNAi sequences.

A. Primer sequences			

Gene	Forward primer, 5′-3′	Reverse primer, 5′-3′	Length of PCR products, bp
VHL	AGAAGGTGGTGGCATTTTTG	AGCAGATGCCAATGCCTTCT	124
HIF-1α	GAAAGCGCAAGTCCTCAAAG	CATACGGTCTTTTGTCACTG	126
HIF-2α	TTGATGTGGAAACGGATGAA	CTCATGGGGTTTTGGGTGAA	110
GAPDH	GAAGGTGAAGGTCGGAGTC	GAAGATGGTGATGGGATTTC	226

B. RNAi sequences			

Gene	Sense oligonucleotide, 5′-3′	Antisense oligonucleotide, 5′-3′	Target gene sequence, 5′-3′

VHL	CGAGCGCGCGCGAAGACUACG	UAGUCUUCGCGCGCGCUCGGU	ACCGAGCGCGCGCGA AGACTACG (98–120 bp)
Negative control	GUACCGCACGUCAUUCGUAUC	UACGAAUGACGUGCGGUACGU	

VHL, Von Hippel-Lindau; HIF, hypoxia-inducible transcription factor; PCR, polymerase chain reaction.
